# Effects of Mind-Body Exercises for Mood and Functional Capabilities in Patients with Stroke: An Analytical Review of Randomized Controlled Trials

**DOI:** 10.3390/ijerph15040721

**Published:** 2018-04-11

**Authors:** Liye Zou, Albert Yeung, Nan Zeng, Chaoyi Wang, Li Sun, Garrett Anthony Thomas, Huiru Wang

**Affiliations:** 1 Department of Sports Science and Physical Education, The Chinese University of Hong Kong, Shatin, Hong Kong, China; 2 Depression Clinical and Research Program, Massachusetts General Hospital, Harvard University, Boston, MA 02114, USA; ayeung@mgh.harvard.edu (A.Y.); GTHOMAS12@mgh.harvard.edu (G.A.T.); 3 Behavioral Health Department, The South Cove Community Health Center, Boston, MA 02111, USA; 4 School of Kinesiology, University of Minnesota-Twin Cities, Minneapolis, MN 55455, USA; zengx185@umn.edu; 5 Department of Physical Education and Sports Science, Jilin University, Changchun 130012, China; chaoyiw@gmail.com; 6 School of Humanities and Social Science, The Chinese University of Hong Kong, Shenzhen 518172, China; sallysuncuhk@gmail.com; 7 Department of Physical Education, Shanghai Jiao Tong University, Shanghai 200240, China

**Keywords:** stroke, rehabilitation, tai chi, mind-body exercise

## Abstract

*Objective*: The effects of stroke are both physical and mental in nature and may have serious implications on the overall well-being of stroke survivors. This analytical review aims to critically evaluate and statistically synthesize the existing literature regarding the effects of mind-body (MB) exercises on mood and functional capabilities in patients with stroke. *Methods*: A structured literature review was performed in both English (PubMed, PEDro, and Cochrane Library) and Chinese (Wanfang and CNKI (Chinese National Knowledge Information Database)) databases. Sixteen randomized controlled trials were considered eligible for meta-analysis. Based on the random effects model, we used the pooled effect size to determine the magnitude of rehabilitative effect of MB exercise intervention on depression, anxiety, activities of daily living, and functional mobility among stroke survivors. The sum PEDro score ranged from five to nine points (fair-to-good methodological quality), but the absence of concealed allocation and blinded assessors were reported in most studies. *Results*: The aggregated results showed that MB exercise intervention is associated with significantly improved ADL (Hedges’ g = 1.31, 95% CI 0.85 to 1.77, *p* < 0.001, *I*^2^ = 79.82%) and mobility (Hedges’ g = 0.67, 95% CI 0.25 to 1.09, *p* < 0.001, *I*^2^ = 69.65%), and reduced depression (Hedges’ g = −0.76, 95% CI −1.16 to −0.35, *p* < 0.001, *I*^2^ = 74.84%). *Conclusions*: as add-on treatments, the MB exercises may potentially improve depression, activities of daily living, and mobility of these post-stroke patients. Future studies with more robust methodology will be needed to provide a more definitive conclusion.

## 1. Introduction

Stroke (also called cerebrovascular disease) is a medical condition in which some regions of the human brain receive insufficient blood supply resulting in cell death and, ultimately, a dysfunctional state [1]. According to the World Health Organization, roughly 15 million people worldwide suffer their first-ever stroke each year, one-third of these sufferers died and another one-third have difficulty in performing activities of daily living (ADL) independently [2]. The Chinese Stroke Prevention Report in 2015 indicated that the prevalence of stroke has dramatically increased among Chinese residents and it has become the leading cause of death in China [3]. Physical therapy and device-assisted rehabilitation methods (e.g., virtual reality, robot-assisted therapy) are commonly used for stroke rehabilitation at the rehabilitation centers, but they are costly and time-consuming. Such rehabilitation methods may not be readily accessible to every post-stroke survivor, particularly those with low socioeconomic status [4,5,6,7].

Given the weakness of conventional rehabilitation and technology-based therapy, exercise training for stroke rehabilitation, as an alternative method, has been investigated and positive results were found [8,9]. However, most exercise programs are physically-based training designed to improve musculoskeletal functions (e.g., mobility, strength, power, and proprioception) of stroke survivors [10,11,12]. Unfortunately, mental health problems (depression, anxiety, and sleep quality) also present among stroke survivors with physical disability [13]. A cross-sectional study by Broomfield [14] indicated that 29% and 23% of stroke survivors had reported anxiety and depression, respectively. These same populations of stroke survivors were associated with increased morbidity and mortality [15]. Thus, it is evident that the effects of stroke are both physical and mental in nature and may have serious implications on the overall well-being of stroke survivors. Therefore, it is urgently needed to establish readily-accessible and cost-effective exercise programs which emphasize both physical and mental rehabilitation for these individuals. 

Implementing mind-body (MB) exercises (tai chi, qigong, and yoga) may provide a solution for this issue given that these exercises are low-cost, low impact, and of moderate-intensity [16,17,18,19]. MB exercises, characterized by a mind-body practice (slow movements and symmetrical postures with musculoskeletal stretching and relaxation, breath control, and mental focus), have recently increased in global popularity [20,21,22,23,24,25]. According to the National Health Interview Survey, Yoga, Tai Chi, and Qigong are ranked as the top three most widely-used complementary therapies among U.S. citizens [26]. As the popularity of MB exercises continue to increase, clinical trials are beginning to investigate the rehabilitative effects of MB exercises for functional capabilities and mental health among stroke survivors [27,28,29,30,31,32]. However, no systematic review to date has been done to critically evaluate the existing literature on this topic. The aim of this study, therefore, was to systematically assess available evidence regarding the effects of MB exercises on these rehabilitative outcomes among stroke survivors. Findings of this review would allow scholars and clinicians to design and develop effective rehabilitation programs for accelerating the recovery process of stroke survivors, while reducing the cost and personnel needs of administering the rehabilitation.

## 2. Methods

To eliminate duplicates, we submitted the present study protocol to the International Prospective Register of Systematic Review for evaluation prior to beginning this project. Since no similar studies had been conducted, the registration number (CRD42018085213) was assigned to this project. To precisely present this systematic review, we used the Preferred Reporting Items for Systematic Review and Meta-analysis (PRISMA) checklist.

### 2.1. Search Strategy

We used both English and Chinese electronic databases for the literature search. The English databases consisted of PubMed, Physiotherapy Evidence Database (PEDro), and Cochrane Library. Given that Tai Chi and Qigong originated from China, two Chinese-language authoritative databases (Wanfang and CNKI) were also searched to maximize the inclusion of relevant literature. With no restriction of publication date, the search terms used for this systematic review included: stroke, cerebrovascular disease, brain ischemia, intracranial hemorrhage, yoga, tai chi/taiji, and qigong. Reference lists of original articles and reviews were manually searched for relevant studies.

### 2.2. Inclusion Criteria and Study Selection

This systematic review only included randomized controlled trials (RCT) published in peer-reviewed journals. To be included, tai chi, qigong, or toga must have been the primary rehabilitation program and its intervention duration must have lasted at least 4 weeks. Additionally, the number of stroke patients should not have been less than 15 in the eligible RCTs. To compute the pooled effect size of individual outcomes (depression, anxiety, sleep quality, mobility, and ADL) of interest, for both MB and control groups, mean and standard deviation at baseline and post-intervention needed to be clearly reported along with the number of participants of each group. The initially-identified studies were considered eligible only if they satisfied all aforementioned criteria. Two investigators (Liye Zou and Nan Zeng) independently screened and identified the eligibility of all studies according to the inclusion criteria. In case disagreement between the two investigators emerged, a third investigator (Chaoyi Wang) was brought in for discussion until the group came to a consensus regarding eligibility. 

### 2.3. Data Extraction from Eligible Randomized Controlled Trials

Data extraction was independently performed by two investigators (Liye Zou and Nan Zeng). Table 1 provides detailed information about the characteristics of all eligible RCTs. This information included reference, study location, participant characteristics (initial sample size and attrition rate, mean age/age range, course of disease, and type of stroke (ischemic/hemorrhage stroke), MB exercise intervention (Training intensity and training mode), outcomes of interest and its testing instruments, and follow-up assessment.

### 2.4. Methodological Quality Assessment

Based on previous studies [33,34], the adapted PEDro scale was used to evaluate the methodological quality of all eligible RCTs. Since blinding of participants and instructor(s) are impractical in a non-pharmaceutical intervention study, we removed these two items. Given that stroke survivors could not be forced to discontinue other mainstream rehabilitation methods (e.g., physical therapy, occupational therapy, or drug treatment), this co-intervention as an item was taken into account. Thus, nine items were finally included in the adapted PEDro scale: (1) randomization; (2) concealed allocation; (3) similar baseline; (4) blinding of assessors; (5) more than 85% retention; (6) missing data management; (7) between-group comparison; (8) point measure and measure of variability; and (9) and co-intervention. Each individual RCT could obtain a maximum of nine points.

### 2.5. Data Synthesis

The pooled effect size of each outcome of interest was computed using the Comprehensive Meta-Analysis Version 2.2 Software (Biostat, Englewood, NJ, USA). We synthesized the study results of individual studies through the random-effect model and 95% confidence interval. We used the *I*^2^ statistic to quantify the degree of heterogeneity (25% = small, 50% = medium, 75% = large). Since there were less than 10 RCTs on each individual outcome of interest, moderator analysis was not performed in this systematic review.

## 3. Results 

### 3.1. Literature Search

**Figure 1:**
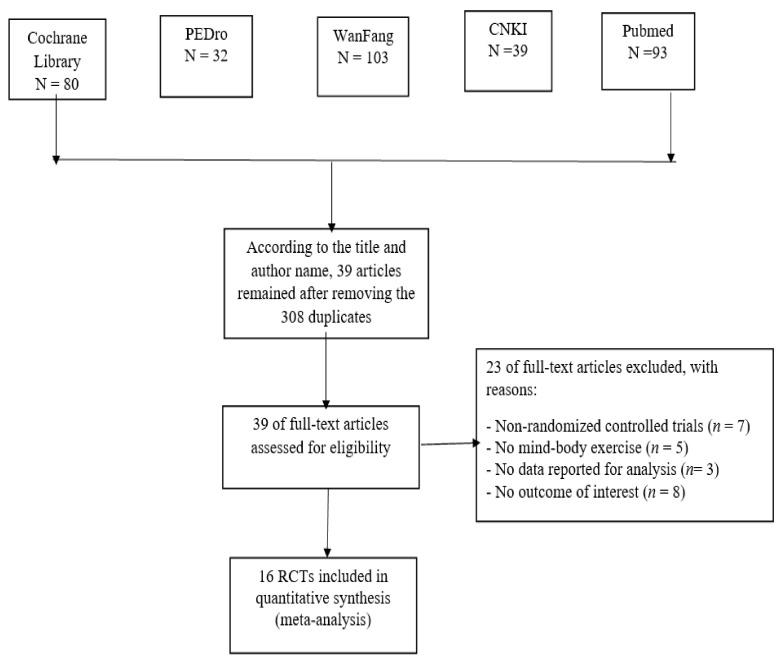
Flowchart showing the retrieval of studies for review (PEDro = Physiotherapy Evidence Database; CNKI = Chinese National Knowledge Information Database).

We found a total of 347 initially-identified records through both English and Chinese databases. According to the title and author name, we removed 308 duplicates, resulting in 39 remaining records. Full-text articles were further evaluated based on the pre-determined inclusion criteria and 23 were removed due to the following reasons: (1) non-RTCs (*n* = 7); (2) no MB exercise (*n* = 5); (3) no interesting outcome (*n* = 8); (4) and unable to extract quantitative data (*n* = 3). Ultimately, this systematic review includes 16 RCTs. The flowchart showing the retrieval of studies for this systematic review is presented in Figure 1.

### 3.2. Study Characteristics

The features of the 16 eligible RCTs are presented in Table 1. These studies were published between 2009 and 2017. A total of 1136 stroke survivors (a mean age from 51.38 ± 14.83 to 77.59 ± 12.33 or age range from 33 to 78), with attrition rate ranging from 8% to 16% (sample size in the RCTs ranged from 24 to 145). Course of disease varied from two weeks to 81.6 months on average. Only 37.5% of the 16 RCTs reported the number of stroke type in each group. For the MB exercise groups (qigong = 1, toga = 1, tai chi = 14), intervention duration ranged from four weeks to 12 weeks, with two to seven sessions per week. A typical session lasted between 15 and 90 min. Training modes in MB exercises were self-practiced, group-based, and mixed method. MB exercise intervention was combined with other components (e.g., usual nursing, general rehabilitation, exercise rehabilitation, usual treatment, drug therapy, physical therapy, or educational program) in 81.25% of the studies selected. Only two studies used follow-up assessment to evaluate the long-term effect of MB exercise on the rehabilitative outcomes. These follow-up assessments occurred at six weeks [30] and 12 months [37], respectively.

### 3.3. Methodological Quality

The PEDro scores of 16 RCTs are presented in Table 2. The sum PEDro scores ranged from five to nine points (fair-to-good methodological quality). Concealed allocation was not used in 81.25% of the 16 RCTs. This is followed by absence of blinded assessors (*n* = 10) and lack of missing data management (*n* = 8). Other points deducted were due to a lack of an attrition rate of more than 15% [30], similar baseline [36] and between-group comparison [39], and absence of point measures and measures of variability [42,43].

### 3.4. Meta-Analysis of Outcome Measured

**Figure 2:**
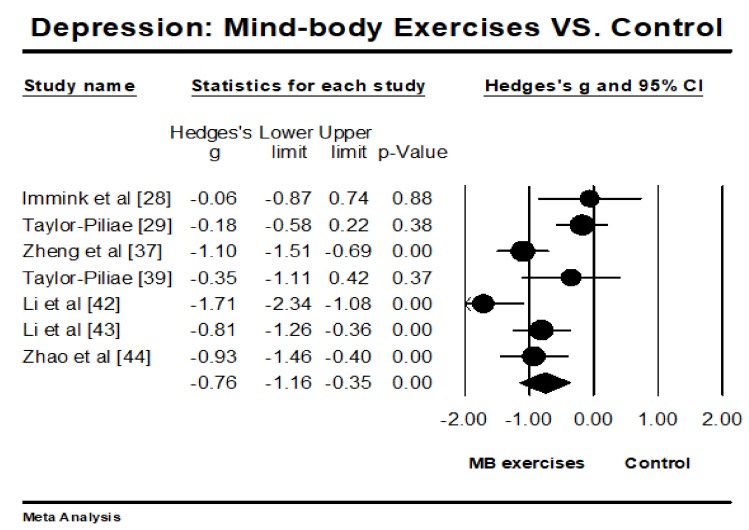
Effect of mind-body exercises on depression. (Circled symbol represents the effect size of each individual study and squared symbol represents an overall/pooled effect size of all studies on each outcome).

The selected RCTs investigated the effects of MB exercises on depression (*n* = 7), anxiety (*n* = 3), and sleep quality (*n* = 3), with lower scores indicating better performance. We used meta-analytic methods to individually synthesize the study findings of each interesting outcome. The aggregated results have shown significant benefit in favor of MB exercises on reducing depression (Hedges’ g = −0.76, 95% CI −1.16 to −0.35, *p* < 0.001, *I*^2^ = 74.84%; Figure 2) and anxiety (Hedges’ g = −1.04, 95% CI −1.33 to −0.74, *p* < 0.001, *I*^2^ = 0%). However, MB exercises did not significantly improve overall sleep quality (Hedges’ g = −0.24, 95% CI −0.56 to 0.08, *p* = 0.14, *I*^2^ = 0%).

**Figure 3:**
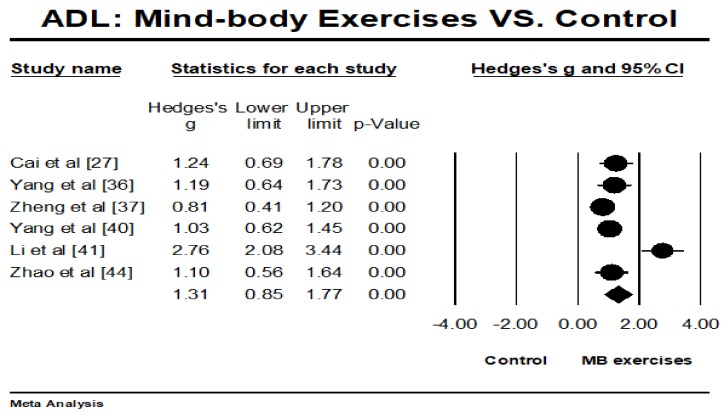
Effect of mind-body exercises on activities of daily living. ADL: activities of daily living. (Circled symbol represents the effect size of each individual study and squared symbol represents an overall/pooled effect size of all studies on each outcome).

**Figure 4:**
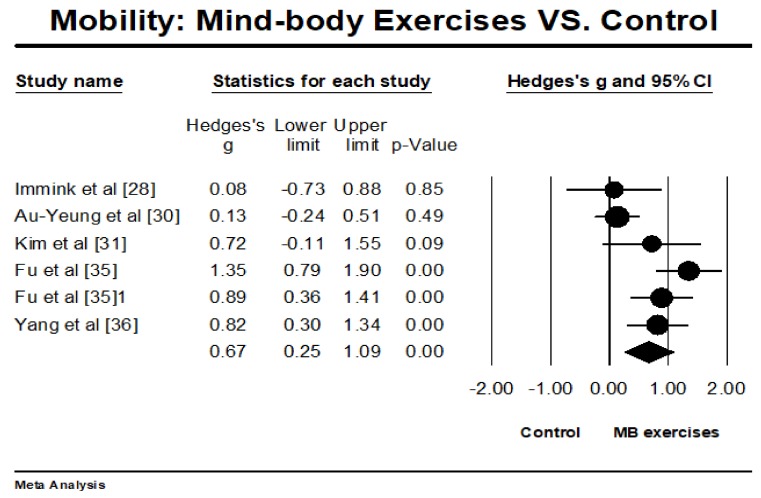
Effect of mind-body exercises on mobility function. (Circled symbol represents the effect size of each individual study and squared symbol represents an overall/pooled effect size of all studies on each outcome).

The selected RCTs investigated the effects of MB exercises on ADL (*n* = 6) and mobility (*n* = 5), with higher positive values indicating better performance. The overall result of the meta-analysis showed that MB exercise intervention was associated with significantly improved ADL (Hedges’ g = 1.31, 95% CI 0.85 to 1.77, *p* < 0.001, *I*^2^ = 79.82%; Figure 3) and mobility (Hedges’ g = 0.67, 95% CI 0.25 to 1.09, *p* < 0.001, *I*^2^ = 69.65%; Figure 4).

## 4. Discussion

The present meta-analytical review was conducted to statistically evaluate the existing literature for the efficacy of MB exercises including tai chi, qigong, and yoga on mood and functional capacities among post-stroke patients. The pooled estimates suggest that MB exercises may have significant benefits in depression, activities of daily life, and mobility among stroke survivors, but positive results on overall sleep quality was not found. There were limited findings for MB exercise effects on anxiety due to the small sample size, however, further study is warranted. The emerging literature has increasingly shown that tai chi/qigong may be a promising adjunct rehabilitative treatment for stroke survivors. To our knowledge, this is the first meta-analysis that included the rehabilitative effects of tai chi and qigong among stroke survivors. The main findings from this systematic review are of great significance for the public health sector since many stroke survivors have varying degrees of depression and loss of functional capacity—both of which affect their mood, functioning, and quality of life [45]. MB exercises can be employed as safe and inexpensive complementary treatments to offer these patients more favorable outcomes.

The underlying mechanisms of how tai chi, qigong, and yoga affect mood and functional capacities in post-stroke patients still remains unclear, though there are several theories that seem plausible. The most notable of these theories is a contemporary concept which suggests these MB exercises enhance physiological proprioception by combining a special state of consciousness with physical movement and breathing techniques, thereby improving and strengthening the overall state of vegetative regulation (homeostasis) [46]. Given that tai chi, qigong, and yoga are physical activities that coordinate complex movements, balance, strengthening, and breathing, the combination of these physical aspects may drive the elicitation of improved functional capacities in patients with stroke. Furthermore, the relaxation and personal integration aspects of these exercises contribute to mindful awareness and personal acceptance [47,48] which may help to establish a more refined mind-body connection, thus strengthening the individual’s homeostatic state. Overall, there are numerous advantages to using MB exercises as adjunctive treatment for stroke survivors. It is accessible to people of all ages and physical strength, easy to learn, and has minimal known side effects. The disadvantage is that when qualified instructors are required to train individuals at the novice level, but there may not be available in some areas.

This study included recently published RCTs in both English and Chinese which used tai chi, qigong, and yoga as the primary intervention. This method is appropriate and important since, thus far, most of the studies on these MB exercises for stroke rehabilitation were conducted in China (including Hong Kong) and were published mostly in Chinese language. By including articles in Chinese, the contributions of researchers on MB exercise studies published in Chinese peer-reviewed journals are acknowledged and the findings are more representative of studies using these MB exercises. Other strengths of this study include the use of a standardized scale to assess the risk of bias for the RCTs, and a recognized meta-analytic method to evaluate the magnitude of the intervention effect of these MB exercise, and the *I*^2^ value to determine the degree of heterogeneity.

We would like to acknowledge the following methodological limitations as they may influence interpretation of these research findings. First, we included tai chi, qigong, and yoga in this review as one large category of MB exercises and summarized their outcomes on post-stroke rehabilitation. While leading researchers in mind-body research propose that tai chi, qigong, and yoga can be considered as mindfulness-based exercises and use similar techniques [49], further studies are needed to confirm that these exercises, in fact, have comparable effects for post-stroke rehabilitation. Second, more than half of the selected RCTs lacked blinding of assessors which might lead to subjectivity and social desirability bias. Third, stroke survivors did not only receive one of these MB exercise interventions in most RCTs, but also simultaneously underwent other rehabilitation programs (e.g., balance training, general rehabilitation, or drug therapy). This co-intervention makes it difficult for researchers to conclude whether the outcomes were due to MB exercises alone, a synergetic intervention effect, or the conventional treatment received by the patients. Nevertheless, the findings of this study provides support for MB exercises as an add-on treatment for post-stroke patients to improve their mood, daily activities, and mobility. Fourth, a variety of interventions were received by control groups which made interpretations of outcomes difficult. Fifth, the frequency and the duration of the MB exercises varied a great deal among different studies. These findings make it difficult to make specific recommendations on the format of the intervention. Sixth, most of the studies were conducted in Asia. It remains unclear whether the results are generalizable to non-Asian populations. In particular, the three included studies that were performed in Western countries [28,29,39] all showed little effect on depression, while the remaining studies showed moderate to large positive effects. These findings raised the question whether the interventions were comparable in different regions. Seventh, studies that report positive or significant results are more likely to be published and outcomes that are statistically significant have greater possibility of being fully reported. Therefore, publication bias and outcome reporting bias might have existed in the included studies, and the effect sizes of MB exercises might have been overestimated. Lastly, although the alpha level was significant in both ADL and mobility, the confidence intervals cross over 1, thus, statistically significant differences in the pooled effect between groups was actually absent. This may be due to the sample size being too small to have enough power to detect a statistically significant results if one exits. Therefore, future studies with large sample sizes may clear things up.

## 5. Conclusions

The findings of this study suggest that, as an add-on treatment, the MB exercises may potentially improve depression, activities of daily living, and mobility of these post-stroke patients. Though there were significant weaknesses in the design of these studies and the outcomes varied in different regions, this should not deter the importance of these findings. Future studies with more robust methodology will be needed to provide a more definitive conclusion; however, the current results appear promising.

## Figures and Tables

**Table 1:** Characteristics of studies selected in this systematic review.

Reference	Participant Characteristics				Mind-Body Intervention			
	ISZ (AT) MB/CG	Mean Age or Age Range	Course of Disease	Ischemic/Hemorrhage	Training Frequency and Length (MB Component)	Training Mode	Outcomes/Instrument	FU
Cai et al. [27], China	60 (0%) 30/30	MB: 60.27 (10.48) CG: 61.27 (7.42)	MB: 29.7 (7.38) wk CG: 28.81 (5.37) wk	MB: 21/9 CG: 24/6	MB: 30 min × 4–5 sessions/wk, 12 wks (Baduanjin qigong) + educational program; CG: educational program	Group	Activities of daily living (Barthel Scale)	No
Immink et al. [28], Austria	25 (12%) 12/13	MB: 56.1 (13.6) CG: 63.2 (17.4)	MB: 81.6 (77.5) M CG: 23.3 (12.5) M	NR	MB: 90 min × 1 session/wk (group-based), 10 wks + 6 × 40 min/session (individual) (yoga); CG: no treatment	Mixed	mobility (2-MWD), depression (GDS), anxiety (STAI)	No
Taylor-Piliae [29], USA	145 (10%) MB: 53 CG1: 44 CG2: 48	MB: 71.5 (10.3) CG1: 69.6 (9.4) CG2: 68.2 (10.3)	MB: 39 (40.2) M CG1: 33 (58.7) M CG2: 38.7 (46.7) M	MB: 33/12 CG1: 32/8 CG2: 30/14 16 unknown	MB: 60 min × 3 sessions/wk, 12 wks (24-style tai chi); CG1: strength and range of movement exercises; CG2: weekly phone call	Group	depression (CES-D), sleep quality (PSQI)	No
Au-Yeung et al. [30], China	136 (16%) 74/62	MB: 61.7 (10.5) CG: 65.9 (10.7)	MB: 54.1 (79.2) M CG: 64.2 (106.4) M	NR	MB: 60 min × 1 session/wk (group) + 60 min × 3 sessions/wk (self-practice), 12 wks (simplified tai chi); CG: General exercises rehabilitation	Mixed	mobility (TUG)	6-wk
Kim et al. [31], Korea	24 (8%) 12/12	MB: 53.45 (11.54) CG: 55.18 (10.2)	NR	NR	MB: 60 min × 2 sessions/wk, 6 wks (simplified tai chi) + (general rehabilitation + physical therapy); CG: general rehabilitation + physical therapy	Group	mobility (TUG)	No
Wang et al. [32], Japan	34 (14.7%) 17/17	MB: 76.53 (9.74) CG: 77.59 (12.33)	NR	NR	MB: 50 min × 1 session/wk, 12 wks (24-style tai chi) + usual treatment; CG: Usual treatment and exercise rehabilitation	Group	sleep quality (PSQI)	No
Fu et al. [35], China	60 (0%) 30/30	MB: 59.7 (7.6) CG: 60.3 (8.4)	Less than 3 months	MB: 13/17 CG: 10/20	MB: 15 min × 6 sessions/wk, 8 wks (24-style tai chi) + General rehabilitation CG: General rehabilitation	Individual	mobility (FAC)	No
Yang et al. [36], China	60 (0%) 30/30	MB: 58 (11.27) CG: 60.07 (7.87)	NR	NR	MB: 15 min × 7 sessions/wk, 4 wks (Tai Chi balance training) + General rehabilitation; CG: General rehabilitation	NR	mobility (FAC), Activities of daily living (Barthel Scale)	No
Zheng et al. [37], China	112 (5%) 56/56	MB: 59 (13) CG: 60 (12)	NR	112/0	MB: 60 min × unclear, 12 wks (tai chi) + General rehabilitation; CG: General rehabilitation	NR	Activities of daily living (Barthel Scale), anxiety (HAMA), depression (HAMB)	12-M
Zhou et al. [38], China	68 (0%) 34/34	65.2 (8.5) for all participants	NR	0/68	MB: unclear × 2 sessions/wk, 4 wks (24-style tai chi) + General rehabilitation; CG: General rehabilitation + drug treatment	NR	anxiety (HAMA)	No
Taylor-Piliae et al. [39], China	28 (11%) 16/12	MB: 72.8 (10.1) CG: 64.5 (10.9)	MB: 58.3 (46.7) M CG: 47.9 (42.5) M	MB: 12/4 CG: 9/3	MB: 60 min × 3 sessions/wk, 12 wks (24-style tai chi); CG: usual treatment	Group	depression (CES-D), sleep quality (PSQI)	No
Yang et al. [40], China	100 (0%) 50/50	MB: 54.3 (13.8) CG: 55.2 (14.6)	MB: 44.7 (18.4) d CG: 42.6 (16.7) d	unable to identify	MB: 45 min × 6 sessions/wk, 4 wks (tai chi balance training); CG: exercise rehabilitation	NR	activity of daily living (Barthel Scale)	No
Li et al. [41], China	67 (0%) 35/32	MB: 56 (5.58) CG: 54 (6.23)	NR	NR	MB: 30–35 min × 5 sessions/wk, 6 wks (tai chi motor imagery) + General rehabilitation; CG: General rehabilitation	Individual	activities of daily living (Barthel Scale)	No
Li et al. [42], China	68 (12%) 36/32	38–76 years	NR	NR	MB: 30 min × 2 sessions/wk, 5 wks (sitting-style tai chi) + usual nursing; CG: usual nursing	Group	Depression (HAMB)	No
Li et al. [43], China	89 (10%) 47/42	33–78 years	2 wks or below	NR	MB: 30 min × 2 sessions/wk, 5 wks (sitting-style tai chi) + usual nursing; CG: usual nursing	Group	Depression (HAMB)	No
Zhao et al. [44], China	60 (0%) 30/30	MB: 53.85 (11.69) CG: 51.38 (14.83)	MB: 40.58 (23.11) d CG: 42.16 (19.82) d	NR	MB: 30 min × 5 sessions/wk, 8 wks (simplified three-form tai chi) + General rehabilitation; CG: General rehabilitation	Group	Depression (HAMB), activities of daily living (Barthel Scale)	No

Note: ISZ = initial sample size, AT = attrition rate; wk. = week; M = month(s); d = day(s); MB = mind-body exercise; CG = control group; FAC = Functional Ambulation classification) HAMDMB = Hamilton Depression Scale; GDS = Geriatric Depression Scale-Short Form; STAI = State Trait Anxiety Inventory; CES-D = the Center for Epidemiologic Studies Depression Scale; PSQI = Pittsburgh Sleep Quality Index; TUG = Timed-up and go test; HAMA = Hamilton anxiety rating scale; HAMDMB = Hamilton Depression Scale; FU = follow-up assessment. NR = not reported; MWD = 2-minute Walk Distance Test.

**Table 2:** Methodological quality for randomized controlled trials and non-randomized controlled studies.

Author [Reference]	Item 1	Item 2	Item 3	Item 4	Item 5	Item 6	Item 7	Item 8	Item 9	Score
Cai et al. [27]	1	0	1	0	1	1	1	1	1	7/9
Immink et al. [28]	1	1	1	1	1	0	1	1	1	8/9
Taylor-Piliae et al. [29]	1	1	1	1	1	1	1	1	1	9/9
Au-Yeung et al. [30]	1	0	1	1	0	1	1	1	1	7/9
Kim et al. [31]	1	0	1	0	1	0	1	1	1	6/9
Wang et al. [32]	1	0	1	1	1	0	1	1	1	7/9
Fu et al. [35]	1	0	1	1	1	1	1	1	1	8/9
Yang et al. [36]	1	0	0	0	1	1	1	1	1	6/9
Zheng et al. [37]	1	0	1	0	1	0	1	1	1	6/9
Zhou et al. [38]	1	0	1	0	1	1	1	1	1	7/9
Taylor-Piliae et al. [39]	1	1	1	1	1	0	0	1	1	7/9
Yang et al. [40]	1	0	1	0	1	1	1	1	1	7/9
Li et al. [41]	1	0	1	0	1	0	1	1	1	6/9
Li et al. [42]	1	0	1	0	1	0	1	0	1	5/9
Li et al. [43]	1	0	1	0	1	0	1	0	1	5/9
Zhao et al. [44]	1	0	1	0	1	1	1	1	1	7/9

Note: Item 1 = randomization; Item 2 = concealed allocation; Item 3 = similar baseline; Item 4= blinding of assessors; Item 5 = more than 85% retention; Item 6 = missing data; management (intention-to-treat analysis); Item 7 = between-group comparison; Item 8 = point measure and measures of variability; Item 9 = Co-intervention (should be either be avoided in the trial design or similar between the index and control groups); 1 = explicitly described and present in details; 0 = absent, inadequately described, or unclear.
